# Frailty Index Predicts All-Cause Mortality for Middle-Aged and Older Taiwanese: Implications for Active-Aging Programs

**DOI:** 10.1371/journal.pone.0161456

**Published:** 2016-08-18

**Authors:** Shu-Yu Lin, Wei-Ju Lee, Ming-Yueh Chou, Li-Ning Peng, Shu-Ti Chiou, Liang-Kung Chen

**Affiliations:** 1 Aging and Health Research Center, National Yang Ming University, Taipei, Taiwan; 2 Institute of Public Health, National Yang Ming University, Taipei, Taiwan; 3 Department of Family Medicine, Taipei Veterans General Hospital Yuanshan Branch, Yilan County, Taiwan; 4 Center for Geriatrics and Gerontology, Kaohsiung Veterans General Hospital, Kaohsiung, Taiwan; 5 Center for Geriatrics and Gerontology, Taipei Veterans General Hospital, Taipei, Taiwan; 6 Health Promotion Administration, Ministry of Health and Welfare, Taipei, Taiwan; The University of Tokyo, JAPAN

## Abstract

**Background:**

Frailty Index, defined as an individual’s accumulated proportion of listed health-related deficits, is a well-established metric used to assess the health status of old adults; however, it has not yet been developed in Taiwan, and its local related structure factors remain unclear. The objectives were to construct a Taiwan Frailty Index to predict mortality risk, and to explore the structure of its factors.

**Methods:**

Analytic data on 1,284 participants aged 53 and older were excerpted from the Social Environment and Biomarkers of Aging Study (2006), in Taiwan. A consensus workgroup of geriatricians selected 159 items according to the standard procedure for creating a Frailty Index. Cox proportional hazard modeling was used to explore the association between the Taiwan Frailty Index and mortality. Exploratory factor analysis was used to identify structure factors and produce a shorter version–the Taiwan Frailty Index Short-Form.

**Results:**

During an average follow-up of 4.3 ± 0.8 years, 140 (11%) subjects died. Compared to those in the lowest Taiwan Frailty Index tertile (< 0.18), those in the uppermost tertile (> 0.23) had significantly higher risk of death (Hazard ratio: 3.2; 95% CI 1.9–5.4). Thirty-five items of five structure factors identified by exploratory factor analysis, included: physical activities, life satisfaction and financial status, health status, cognitive function, and stresses. Area under the receiver operating characteristic curves (*C*-statistics) of the Taiwan Frailty Index and its Short-Form were 0.80 and 0.78, respectively, with no statistically significant difference between them.

**Conclusion:**

Although both the Taiwan Frailty Index and Short-Form were associated with mortality, the Short-Form, which had similar accuracy in predicting mortality as the full Taiwan Frailty Index, would be more expedient in clinical practice and community settings to target frailty screening and intervention.

## Introduction

In contrast with major medical advances in extending life expectancy during the twentieth century, a core focus of health care services nowadays is pursuing enhanced quality of life. Though life expectancy almost doubled during the last century, longevity was gained at the expense of loss of physical function.[1] Ironically, by changing the global burden from communicable diseases and premature death, to non-communicable disease and related chronic disability, the triumph of clinical medicine and public health has presented a huge challenge.[2, 3] Disability usually results from progressive functional decline, which is characterized by reduced physical capacity, increased vulnerability to stressors, and disrupted multi-system homeostasis: collectively, frailty.[4, 5] Importantly, appropriate intervention programs can reverse frailty and reduce disability;[6–8] therefore, frailty prevention and intervention has become an important focus for promoting health among older people.[9]

Several operating definitions of frailty have been developed; these include phenotypic approaches, like the Cardiovascular Health Study (CHS),[4] and index definitions, such as the Frailty Index (FI),[10] and the Kihon checklist.[11] Both kinds of approach predict adverse outcomes such as falls, hospitalization and mortality.[12–17] Among these definitions, FI was developed based on the concepts of aging as a process of accumulating deficits and quantifying vulnerability for older adults.[18] Unlike the CHS definition, FI directly measures health deficits across the domains of physical, social and cognitive function,[19] and reflects physiological aging, to better predict mortality.[20, 21] These key characteristics have important public health implications: 1) The multi-dimensional FI approach suggests feasible preventive measures and potential interventions; and 2) biological age may be an optimal metric for targeting medical therapeutic interventions and public health programs. Similar to QRISK2 in predicting cardiovascular risk, which encouraged people to live more healthily by showing how mortality risk changed if individual risk was modified,[22] FI may also help to foster healthy aging among older people. In a recent study of nine nursing homes, FI also demonstrated the potential to support health economic evaluations for better allocating healthcare resources, and provided insights for public health.[23]

Lifestyle and cultural context may influence each individual’s different domains of accumulated deficits, which may contribute to FI heterogeneity across different populations and geographies.[16, 24–28] The association between FI and mortality has been validated in Canada,[16] Italy,[24] the United Kingdom,[25] the Netherlands,[26] Hong Kong,[27] and China,[28] but not yet in Taiwan. Besides establishing a FI in Taiwan, identifying its potential structure factors is very important in developing national active-aging initiatives. Therefore, the main aims of this study were to construct a Taiwan Frailty Index (TwFI), and to use exploratory factor analysis to ascertain structure factors for a Taiwan active-aging scheme.

## Methods

### Participants and study design

Study data were excerpted from the second wave of The Social Environment and Biomarkers of Aging Study (SEBAS), in 2006;[29] this population-based cohort study had used multistage proportional-to-size sampling to represent all Taiwanese aged 53 years and older, with the intention of investigating the association between biological, psychological, and social aspects of senior health. SEBAS design, participants recruitment, and data collection are detailed elsewhere.[29] Briefly, from 1,659 potential participants invited between August 2006 and January 2007, 1,284 (77.4%) who responded were interviewed face-to-face at home by well-trained research nurses, having first provided written consent.

### Ethics statement

The observational design and reporting format follow STROBE (Strengthening the Reporting of Observational Studies in Epidemiology) guidelines.[30] The Institutional Review Boards at Princeton University, NJ, and Georgetown University, Washington DC, USA, and the Joint Institutional Review Board of Taiwan approved the study protocol (06-044-C), and written informed consents were obtained from all of the participants.

### Mortality ascertainment

All participants were followed from their interview date until 31 December 2010. Data on the dependent variable of death were acquired from the national death registry held by the Ministry of Health and Welfare, Taiwan.

### Selecting variables to construct a Frailty Index

SEBAS data included: demographic information; subjective health evaluation; chronic conditions; physical function; health behavior; mental health (depressive symptoms, self-mastery and cognitive function); social participation; and life-related stress. Based on the principles of FI development, [31] a consensus meeting of geriatric medicine experts selected 153 candidate SEBAS data items, which included multimorbidity, physical function, mental conditions, social participation and socioeconomic status; having excluded items with > 10% of values missing the resulting TwFI comprised 139 health (deficit) factors (S1 Table).

### Health deficits coding

All health deficits were denoted by numeric values between zero and one, with the same weighting, to evaluate their extent; for instance, binary variables such as “Do you have physician-diagnosed diabetes mellitus? (yes/no)” would be coded as ‘0’ if negative, or ‘1’ denoting affirmation of that health deficit. A value of 0.5 indicated a single intermediate response (eg, sometimes or maybe). Variables quantified by four or five-point Likert scale, were recoded from ‘0’ to ‘1’ accordingly, with larger values indicating worsening health deficits. For instance, one of most common subjective health evaluation questions “How would you grade your health status? Excellent, Very Good, Good, Fair, Poor?” would be recoded: Excellent = ‘0’; Very Good = ‘0.25’; Good = ‘0.5’; Fair = ‘0.75’; Poor = ‘1’). FI was calculated as:

$$\text{Frailty Index}=\frac{\text{Summed health deficits scores}}{\text{Total health deficits items}}$$

### Statistical analysis

Numerical variables were expressed as mean ± standard deviation, categorical variables as number (percentage). Mean imputation was used to manage missing values. Mean and 95% confidence intervals (CI) were obtained and plotted across 5-year interval age groups. Kaplan–Meier survival analysis with log-rank test was used to examine the equality of TwFI tertiles. Cox regression analysis was used to assess the association between TwFI and overall mortality. Schoenfeld residuals were used to test proportionality assumptions of Cox proportional hazard models. First, TwFI multiplied by 100 (unit = 0.01) was used as the continuous variable, to maximize statistical efficiency; then its tertiles were used to assess the relationship between TwFI and mortality risk.

Exploratory factor analysis is widely used to simplify the order of interrelated measures, and to investigate the possible structure of underlying factors.[32] This study used exploratory factor analysis to reduce the number of variables and to identify the numbers of latent constructs and the underlying factor structures. Kaiser-Meyer-Olkin measure of sampling adequacy provided an index to assess the appropriateness of factor analysis, with a high value indicating that samples were suitable for factor analysis because correlation between pairs of variables could be explained by other variables; a Kaiser-Meyer-Olkin value ≥0.6 indicated the appropriateness of principal axis factoring. In extracting principal axis factors, Cattell’s scree test and total variance were used to determine the smallest number of structure factors able to explain most of the variation of all items. Factors with eigenvalues > 1.0 were extracted for rotation according to the Varimax orthogonal rotation technique,[33] with factor loadings of ≥ 0.5 defined as relevant.[34] The new variables obtained from exploratory factor analysis constituted a short-form TwFI (TwFI-SF).

Plotting sensitivity against (1 minus specificity) at all possible threshold settings yields a receiver operating characteristic (ROC) curve; the area under this curve, termed *C*-statistic, indicates the discriminative ability of diagnostic tests. Differences in *C*-statistics between TwFI and TwFI-SF were analyzed by the method of DeLong et al.[35]

A *p*-value from two-sided tests < 0.05, and 95% CIs not spanning the null hypothesis values were considered statistically significant. All analyses were performed using the SAS statistical package, version 9.4 (SAS Institute, Inc., Cary, NC, USA).

## Results

**Fig 1:**
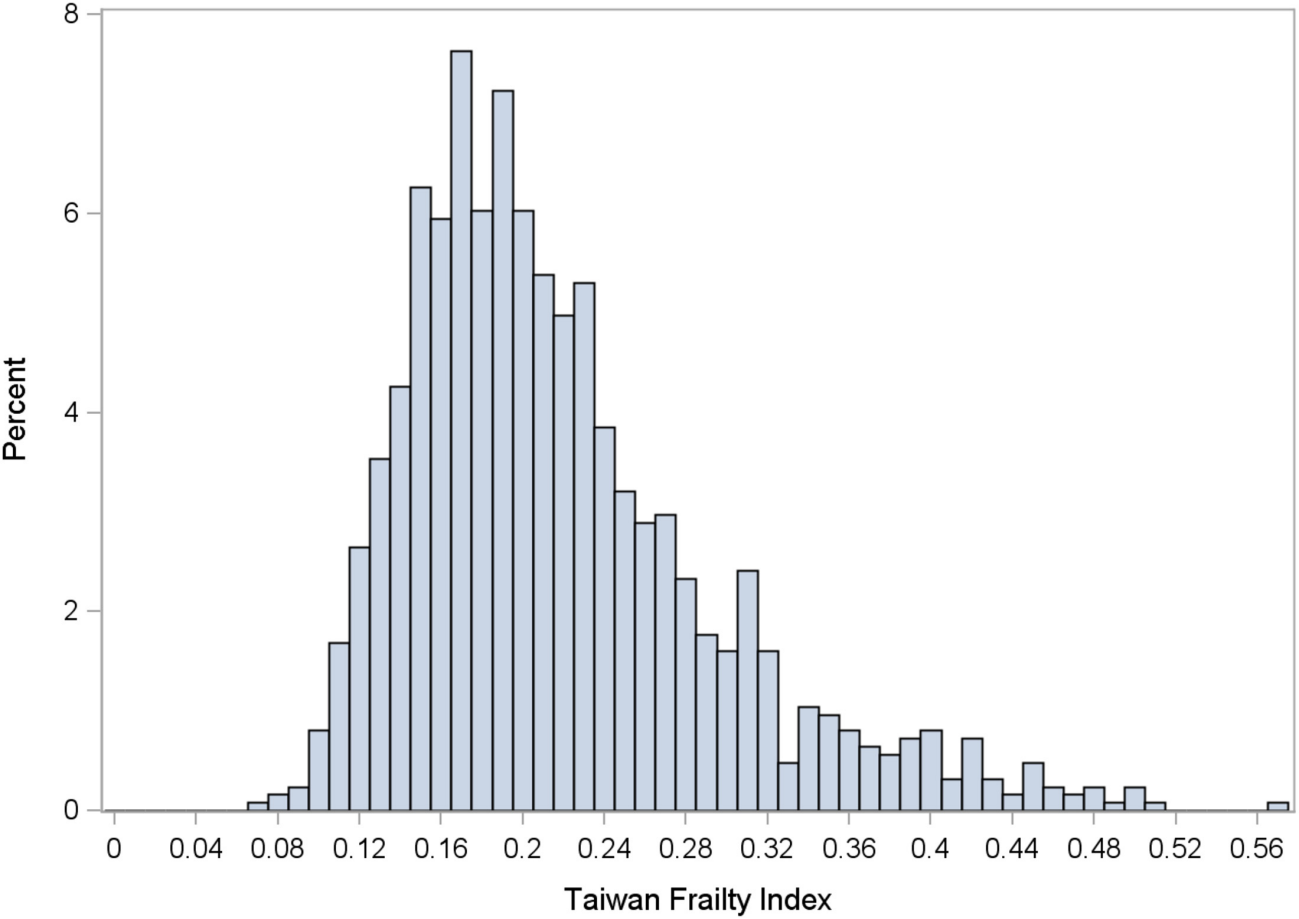
Distribution of Taiwan Frailty Index.

**Fig 2:**
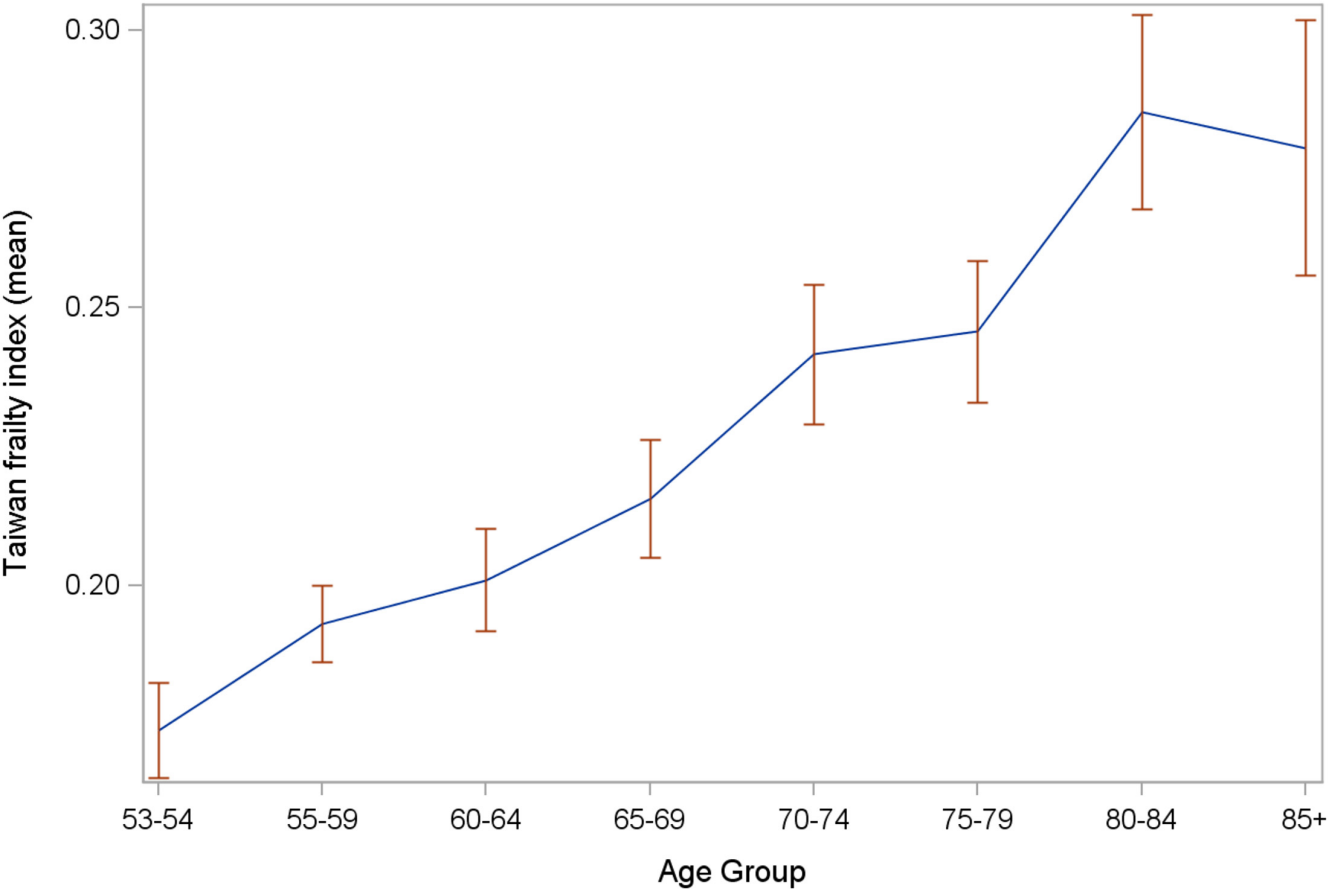
Means and 95% confidence intervals of Taiwan Frailty Index across different age groups.

The analytic cohort comprised 1,245 participants (mean age 66.0 ± 10.0 years, 47.5% women), after excluding 39 (3.0%) with incomplete data. During the study period, with average follow-up of 4.3 ± 0.8 years, 139 deaths occurred (11%, 2.7 per 100 person-years at risk). The constructed TwFI had a median value of 0.2 (range 0.08–0.57) in the target population, and a right-skewed distribution (Fig 1); mean TwFI increased with age between 53 and 79 years, but decreased above age 80 (Fig 2).

**Fig 3:**
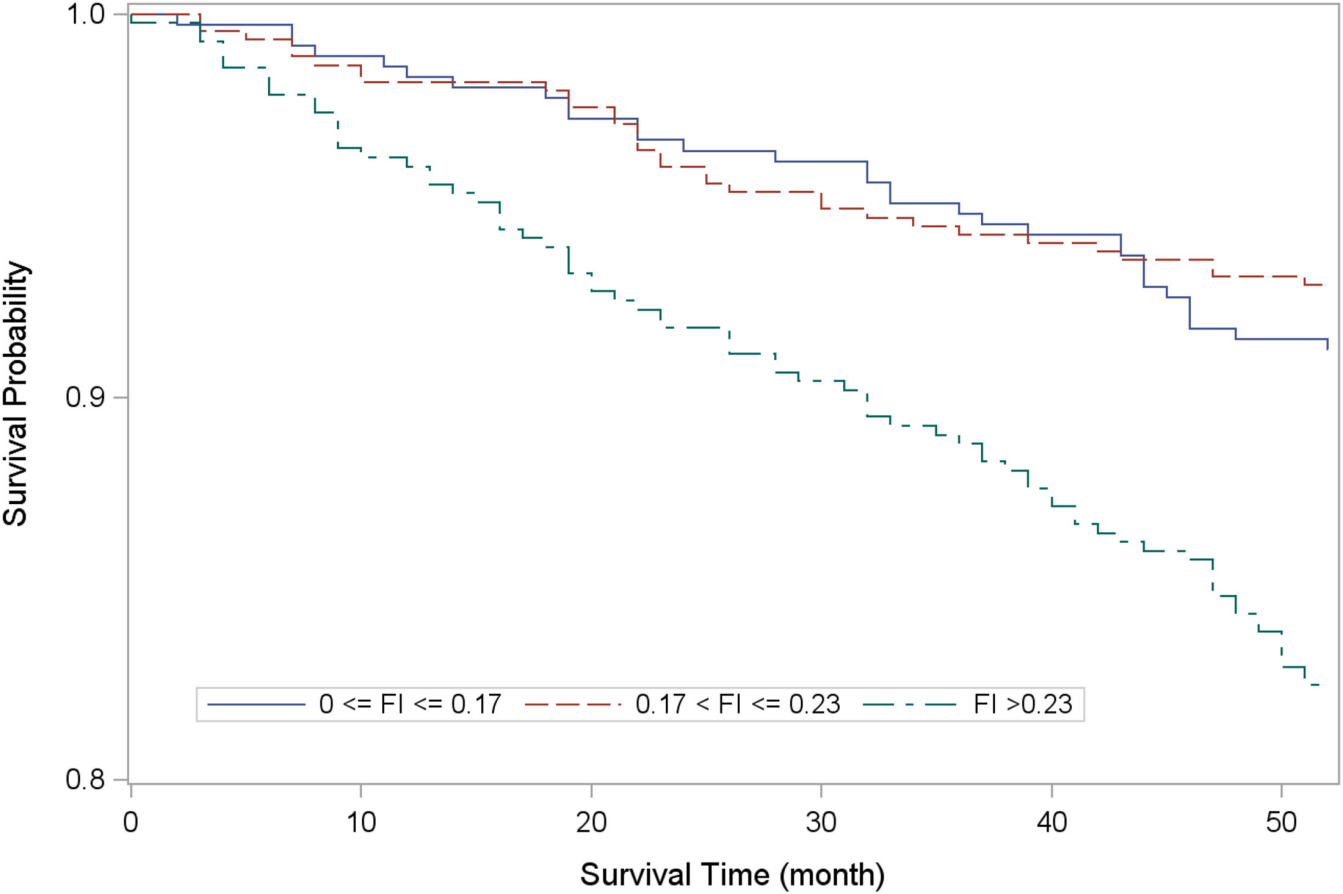
Kaplan-Meier survival analysis by tertile level of Taiwan Frailty Index.

Table 1 summarizes the participants’ demographic characteristics by tertiles; there were proportionally more women, hospitalizations in the past year, and multimorbidity from the lowest through the highest TwFI tertile level. Kaplan-Meier analysis showed significantly lower survival probability among the lowest TwFI tertile relative to the others (Fig 3). When TwFI was considered a continuous variable, age- and sex-adjusted Cox regression analysis showed that mortality risk increased by 3.9% for each 1.0% increment in TwFI during follow-up (Hazard ratio [HR]:1.04; 95% CI 1.02–1.06; *p* < 0.001). Compared to the lowest tertile, the uppermost (TwFI > 0.23) had significant higher mortality risk (HR 1.54; 95% CI 1.01–2.35; p = 0.047), whereas there was no statistical significance compared with the middle tertile– 0.17 < TwFI ≤ 0.23 (HR 0.72; 95% CI 0.44–1.18; *p* = 0.190). When frailty was considered as FI>0.2, a value of cut-off points based on median of the sample and previous literatures, [16,36,37] risk for mortality was similar (HR 1.94;95% CI 1.34–2.79). The association between TwFI and survival was also examined by age groups (<65 vs. ≥65 years). Limited to statistical power, the association of TwFI and mortality (highest tertile vs. lowest tertile) did not reach statistical significance among both younger (HR:1.4 95%CI 0.6–3.3, p = 0.408) and older group (HR:1.6 95%CI 1.0–2.6, p = 0.069).

**Table 1:** Participant characteristics by tertile level of Taiwan Frailty Index.

Variables: data show mean ± standard deviation, or number (%)		Frailty Index Tertile			
		0.0 to 0.17 (n = 365)	> 0.17 to 0.23 (n = 452)	> 0.23 (n = 428)	*p* value
Age (years)		64.1 ± 9.0	65.3 ± 9.6	68.4 ± 10.4	<0.001
Sex	Men	215 (58.9)	253 (56)	211 (49.3)	0.020
	Women	150 (41.1)	199 (44)	217 (50.7)	
Frailty Index		0.14 ± 0.02	0.2 ± 0.02	0.3 ± 0.07	<0.001
Smoking in past 6 months	No	284 (77.8)	361 (79.9)	355 (82.9)	0.200
	Yes	81 (22.2)	91 (20.1)	73 (17.1)	
Hospitalization in past year	No	357 (97.8)	403 (89.2)	307 (71.7)	<0.001
	Yes	8 (2.2)	49 (10.8)	121 (28.3)	
Health examination in past year	No	243(66.6)	316(69.9)	318(74.3)	0.057
	Yes	122(33.4)	136(30.1)	110(25.7)	
Multimorbidity	No (< 2 diseases)	288 (78.9)	193 (42.7)	84 (19.6)	<0.001
	Yes (≥ 2 diseases)	77 (21.1)	259 (57.3)	344 (80.4)	
Satisfaction of current living situation					<0.001
	Very satisfied	83(22.7)	64(14.2)	47(110	
	Satisfied	218(59.7)	260(57.5))	165(38.6)	
	Average	64(17.5)	107(23.7)	155(36.2)	
	Dissatisfied	0	21(4.7)	54(12.6)	
	Very dissatisfied	0	0	7(1.6)	
Subjective rated health					<0.001
	Excellent	104(28.5)	37(8.2)	10(2.3)	
	Good	130(35.6)	97(21.5)	35(8.2)	
	Average	122(33.4)	252(55.8)	144(33.6)	
	Not so good	9(2.5)	64(14.2)	187(43.7)	
	Poor	0	2(0.4)	51(11.9)	
Stress on family member's health					<0.001
	No	315(86.3)	331(73.2)	258(60.3)	
	Some stress	45(12.3)	98(21.7)	104(24.3)	
	A lot of stress	3(0.8)	19(4.2)	53(12.4)	
Stress on family member's finance					<0.001
	No	334(91.5)	345(76.3)	269(62.9)	
	Some stress	28(7.7)	88(19.5)	88(20.6)	
	A lot of stress	1(0.3)	14(3.1)	58(13.6)	

**Fig 4:**
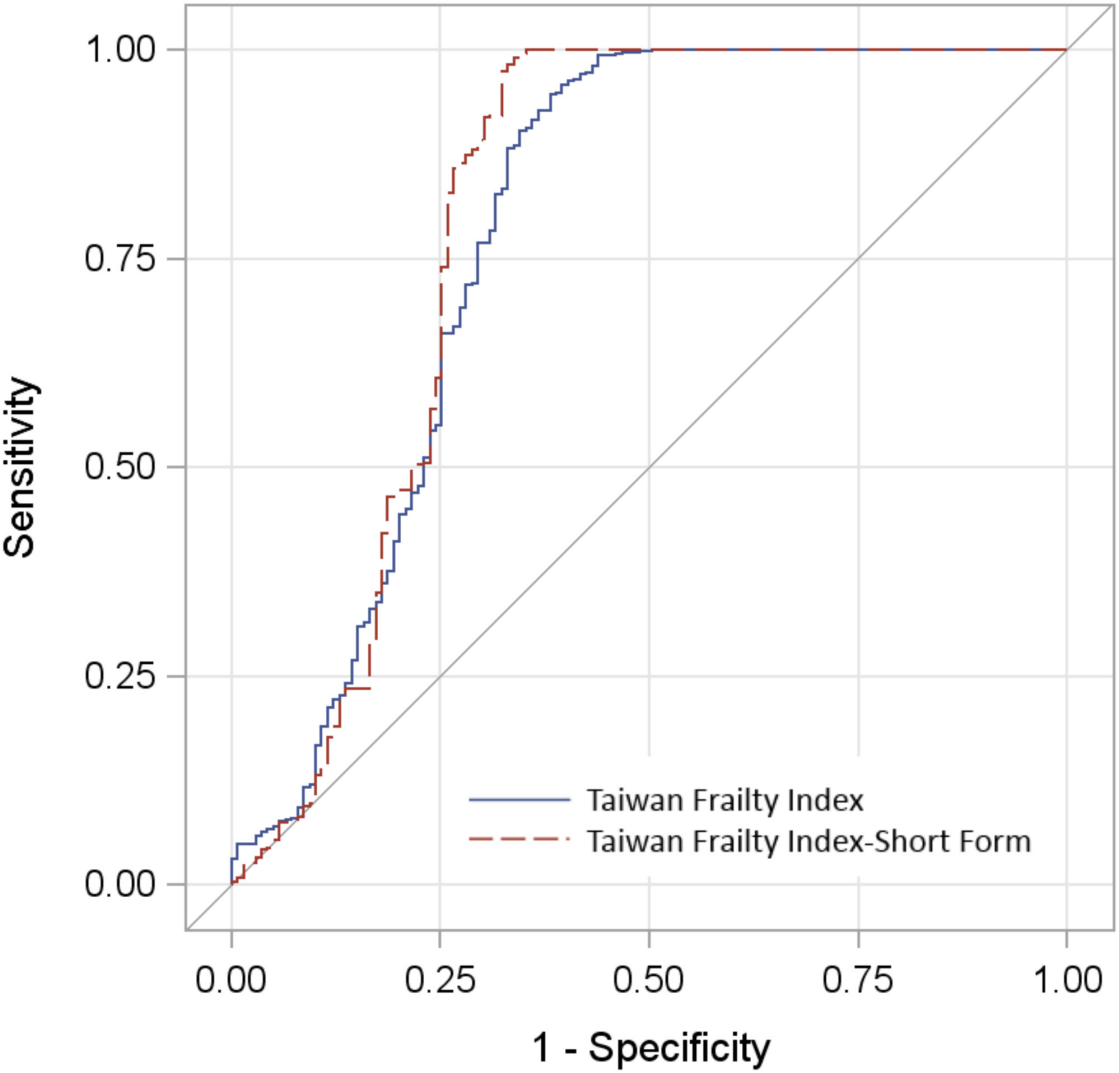
Comparison of *C*-statistics of Taiwan Frailty Index and Taiwan Frailty Index Short-Form.

The Kaiser-Meyer-Olkin measure of adequate sampling prior to exploratory factor analysis was 0.899, indicating that factor analysis was appropriate. In extracting principal axis factors, the Cattell’s scree test identified five solutions, designated: Factor I (Physical activity); Factor II (Life satisfaction & financial status); Factor III (Health status); Factor IV (Stress); and Factor V (Cognitive function). Table 2 shows the TwFI-SF with these 35 items and their loading factors. Loading factor were generally higher in physical activity(Factor I) and similar in other three Factors, which might imply the major contribution of physical activity for FI. In ROC analysis (Fig 4), the *C*-statistics of TwFI and TwFI-SF were 0.78 (95% CI 0.73–0.84) and 0.80 (95% CI 0.74–0.86), respectively, with no statistically significant difference between them.

**Table 2:** TwFI-SF factors and loading factors by exploratory factor analysis with principal axial factoring and orthogonal varimax rotation.

Factor I: Physical activity		Factor II: Life satisfaction & financial status		Factor III: Health status		Factor IV: Stress		Factor V: Cognitive function	
Item (loading factor)		Item (loading factor)		Item (loading factor)		Item (loading factor)		Item (loading factor)	
Standing continuously for 15 minutes	(0.64)	Satisfaction of current living situation	(0.52)	Multimorbidity	(0.68)	Stress on one’s own finances	(0.52)	Orientation to time (year)	(0.65)
Raising both hands over head	(0.52)	Happy	(0.50)	Subjective rated health	(0.50)	Stress on family member’s health	(0.51)	Orientation to time (month)	(0.75)
Grasping or turning objects with fingers	(0.64)	Life goes well	(0.49)	Pain	(0.50)	Stress on family member’s finance	(0.55)	Orientation to time (date)	(0.69)
Walking 200–300 meters	(0.55)	Meeting living expenses	(0.51)	Health status evaluated by observers	(0.50)	Stress on family member’s job	(0.53)	Orientation to time (day of the week)	(0.51)
Climbing 2–3 flights of stairs	(0.55)	Helpless in dealing with problems of life	(0.50)					Orientation (current President)	(0.58)
Buying personal items	(0.70)	Subjective socioeconomic status	(0.50)					Orientation (former President)	(0.55)
Managing money/paying bills	(0.60)								
Riding bus/train by yourself	(0.57)								
Doing light tasks at home	(0.70)								
Bathing	(0.83)								
Dressing	(0.85)								
Eating	(0.63)								
Getting out of bed/standing up/sitting in chair	(0.88)								
Moving around the house	(0.89)								
Toilet	(0.86)								

TwFI-SF, Taiwan Frailty Index Short-Form

## Discussion

This study used a nationally representative population-based cohort to construct a Frailty Index for Taiwan and ascertained the five-factor structure of the TwFI-SF for clinical practice and public health programs; these factors included physical activity, life satisfaction and finance status, health status, stress, and cognitive function. TwFI was significantly associated with all-cause mortality, and the TwFI-SF had similar discrimination ability for predicting mortality. These findings are not only compatible with previous reports, but also simplified the FI through factor analysis; moreover, factor analysis clearly identified important domains for active-aging policies and health promotion for older people in Taiwan.

The right-skewed distribution of TwFI and median value 0.2, were similar to previous studies.[25, 26] Likewise, significant association with age, was congruent with results from other countries.[16, 27, 28] In a study of 2,195 community-dwelling middle-aged adults, 10-year cardiovascular mortality risk increased by 61% per 0.1 unit increment of FI.[13] Among 951 Netherlands adults with intelligence-deficits, those with FI greater than 0.2 had substantially increased risk of 3-year mortality.[36] Canadian investigators reported that 10-year mortality risk rose by 1% to 8% with each incremental FI deficit.[16] These studies affirm that FI predicts all-cause and cause-specific mortality among people with different health status. Mortality risk in the SEBAS cohort increased by 4% per 0.01 unit increase in FI. For health promotion, frailty intervention and disability prevention programs, an optimal FI cut-off is needed; many previous studies have defined frailty as an FI of ≤0.2.[16, 36, 37] The TwFI cut-off of 0.23 determined in this study was similar to that in the Canadian Study of Health and Aging, which found that people with FI greater than 0.21 had less than 5% chance of having “robust” health for their age,[37]

Although this study developed TwFI according to standard procedures,[31] using 139 items may limit its feasibility in daily practice. Mitnitski et al, proposed that FI composed of more than 30 randomly selected health deficits was an adequate proxy for health status in older adults.[38] We used exploratory factor analysis to investigate latent structure and reduce factors, to develop a 35-item TwFI-SF, which identified five factors, designated as physical activity, life satisfaction & financial status, health status, stress and cognitive function. Similar to the British Women’s Heart and Health Study,[25] physical activity, health status, and cognitive function were three major structure factors; however, this study found life satisfaction and economic status, as well as stress, to also be important metrics to evaluate older people’s health. ROC analysis showed both TwFI and TwFI-SF to have good and similar predictive ability for all-cause mortality, which supports using the TwFI-SF as a proxy for health of older Taiwanese, and to evaluate the effectiveness of public health programs. Moreover, ROC analysis disclosed that sensitivity reached 1 before 1 minus specificity reached 0.5, and that TwFI-SF was more sensitive than TwFI; in other words, notwithstanding similar accuracy in predicting mortality, TwFI-SF had even higher detection ability, and lower probability of erroneously predicting survival as death.

Though most studies consider each FI item as having equal-weight, Kamaruzzaman et al, have argued that appropriate weighting is necessary, due to the different impact of catastrophic disease and physical activities on frailty;[25] contrarily, others contest that the total number of items already reflects the severity of health deficits, so no further adjustment is needed.[16] Furthermore, simple unweighted FI has the merit of being easily-calculated and more expedient in public health programs.

This study has important implications for policymakers and healthcare professionals. First, frailty is an intermediate state of disability, and early detection of frailty promotes early intervention to reduce associated adverse outcomes. TwFI-SF is suitable for assessing older people’s health status. Moreover, repeated measurements of TwFI-SF over time may facilitate monitoring the effect of intervention programs or public health policies. Second, the major endeavor of five-factor structure TwFI-SF identified feasible domains for devising frailty interventions, disability prevention and other public health programs. Nevertheless, this study had several limitations. First, TwFI and phenotypic definitions of frailty were not compared, due to limited SEBAS data. Second, sex-specific and cause-specific mortality analysis were not possible, due to limited numbers of events. Third, TwFI was constructed based on a face-to-face interview; the appropriateness and feasibility of self-administering this questionnaire therefore remains unclear, and deserves further investigation.

## Conclusion

TwFI and TwFI-SF effectively predict all-cause mortality among middle-aged and older people in Taiwan; 35-item TwFI-SF was as effective as TwFI comprising 139 items, and also had better discrimination ability. TwFI-SF should be considered an expedient evaluation and monitoring tool for active aging programs and policy-making processes.

## Supporting Information

S1 Table List of variables recorded by the Social Environment and Biomarkers of Aging Study used to construct the 139-item Taiwan Frailty Index. (DOCX)
