# Supplementary material for: Frailty Index Predicts All-Cause Mortality for Middle-Aged and Older Taiwanese: Implications for Active-Aging Programs
Source: PLoS One. 2016 Aug 18;11(8):e0161456. doi: 10.1371/journal.pone.0161456 (PMC4990295; doi:10.1371/journal.pone.0161456)
Supplement: S1 Table — (DOCX) [file pone.0161456.s001.docx]

**S1 Table. List of variables recorded by the Social Environment and Biomarkers of Aging Study used to construct the 139-item Taiwan Frailty Index**

1. Marital status
2. Living alone
3. How do you like house where you live
4. What do you think of neighborhood and area where you live
5. Satisfaction of current living situation
6. Do you have any living children now
7. At least see or speak to one neighbor or friend
8. Too many demands from close friend or relatives
9. Subjective rated health
10. Subjective health compared to 1 year ago
11. Subjective health compared to people at your age
12. Hypertension
13. Diabetes mellitus
14. Heart disease
15. Stroke
16. Cancer
17. Lower respiratory disease
18. Arthritis or rheumatism
19. Gastric ulcers and gastric problems
20. Liver and gall bladder diseases
21. Hip fracture
22. Cataract
23. Kidney disease
24. Gout
25. Osteoarthritis of spine
26. Osteoporosis
27. Multimorbidity
28. Fall or accidental injury
29. Any difficulty standing continuously for 15 minutes
30. Any difficulty standing continuously for 2 hours
31. Any difficulty squatting
32. Any difficulty raising both hands over head
33. Any difficulty grasping or turning objects with fingers
34. Any difficulty lifting 11–12 kilograms
35. Any difficulty running 20–30 meters
36. Any difficulty walking 200–300 meters
37. Any difficulty climbing 2–3 flights stairs
38. Any difficulty buying personal items
39. Any difficulty managing money/paying bills
40. Any difficulty riding bus/train by yourself
41. Any difficulty doing physical work at home
42. Any difficulty doing light tasks at home
43. Any difficulty making phone calls
44. Any difficulty bathing
45. Any difficulty dressing
46. Any difficulty eating
47. Any difficulty getting out of bed/standing up/sitting in chair
48. Any difficulty moving around the house
49. Toilet
50. Presence of body pain
51. Duration of moderate/severe pain during past year
52. Subjective sleep quality
53. Daytime drowsiness
54. Chi kung
55. Tai chai
56. Meditation
57. Yoga
58. Other folk activities similar to chi kung
59. Regular exercise
60. Smoking
61. Poor appetite
62. Exhaustion
63. Restless sleep
64. Depressed
65. Lonely
66. Perceive other people are unfriendly
67. Sad
68. Low energy
69. Happy
70. Life goes well
71. Language: naming watch
72. Language: naming pencil
73. Repetition
74. Orientation to place
75. Orientation to time (year)
76. Orientation to time (month)
77. Orientation to time (date)
78. Orientation to time (day of the week)
79. Orientation (current President)
80. Orientation (former President)
81. Backward repetition
82. Hospitalization in past year
83. Emergency Department visit in past year
84. Health examination
85. Playing chess or Mahjong
86. Chatting with neighbors or friends
87. Outdoor activities
88. Group activities, such as dancing, singing or karaoke
89. Neighborhood association (such as women's association, arts & craft class, etc.)
90. Recall function
91. Any difficulty meeting living expenses
92. Expect more good things to happen to me than bad
93. Stress on one’s own health
94. Stress on one’s own finances
95. Stress on one’s family relationship
96. Stress on family member’s health
97. Stress on family member’s finances
98. Stress on family member’s job
99. Stress on family member’s marriage
100. I have little control over the things that happen to me
101. What happens to me in the future mostly depends on me
102. No way I can solve some of the problems I have
103. There is little I can do to change many of the important things in my life
104. I can do just about anything I really set my mind to
105. I often feel helpless in dealing with the problems of life
106. Sometimes I feel that I am being pushed around in life
107. Argument/disagreement with anyone in last 24 hours
108. Anything else happen in last 24 hours that was stressful
109. Current residence/community environment is safe or not
110. Victims of crime (oneself)
111. Victims of crime (family members)
112. Fraud events (oneself)
113. Fraud events (family members)
114. Worried about the security of life and assets
115. Worried about the tensions between Taiwan and Mainland China
116. Worried about war/social chaos/political changes in future
117. Death of spouse
118. Divorce
119. Death of close family member
120. Jail
121. Death of close friend
122. Change in financial state (income decrease)
123. Investment/credit difficulties
124. Mortgage or loan
125. Change in health of family member
126. Marital reconciliation
127. Change in residence
128. Loss of/damage to personal property
129. Serious argument with relative or friend
130. Involved in a law suit
131. Changed jobs
132. Lost job
133. Retirement
134. Major personal injury/illness
135. Natural disaster
136. Immediate family member, romantic partner, or very close friend died because of an accident, homicide, or suicide
137. Subjective socioeconomic status
138. Health status evaluated by observers
139. Vision impairment
